# Global research progress of nanomedicine and colorectal cancer: a bibliometrics and visualization analysis

**DOI:** 10.3389/fonc.2024.1460201

**Published:** 2024-12-06

**Authors:** Siyu Tian, Min Chen

**Affiliations:** Proctology Department, Affiliated Hospital of Chengdu University of Traditional Chinese Medicine, Chengdu, China

**Keywords:** nanomedicine, colorectal cancer, bibliometrics, photodynamic therapy, immunogenic cell death, tumor microenvironment

## Abstract

**Background:**

Surgery and chemoradiotherapy are the main clinical treatment methods for colorectal cancer (CRC), but the prognosis is poor. The emergence of nanomedicine brings bright light to the treatment of CRC. However, there has not been a comprehensive and systematic analysis of CRC and nanomedicine by bibliometrics.

**Methods:**

We searched the Web of Science Core Collection database (WOSCC) for relevant literature published from 2011 to 2024. We used VOSviewer and Citespace to analyze countries, institutions, authors, keywords, highly cited references, and co-cited references.

**Results:**

3105 pieces of literatures were included in the research analysis, and PEOPLES R CHINA and the USA took the leading position in the number of papers published and had academic influence. The Chinese Academy of Sciences posted the most papers. The most prolific scholar was Abnous Khalil. The level of economic development is inversely proportional to the number of cases and deaths of colorectal cancer. Nanoparticles (NPs), the nanomedical drug delivery system (NDDS) is a hot topic in the field. Photodynamic therapy (PDT), immunogenic cell death (ICD), tumor microenvironment (TEM), folic acid, and pH are the cutting edge of the field.

**Conclusion:**

This paper introduces the research hotspot, emphasis, and frontier of CRC and nanomedicine, and points out the direction for this field.

## 1 Introduction

Colorectal cancer (CRC) represents approximately 10% of global cancer incidence and mortality rates. More than 900,000 deaths per year, making CRC the second deadliest cancer in the world (1). By 2035, there will be 2.5 million new cases of CRC worldwide (2). Demonstrating that colorectal cancer is a disease of paramount global health significance. Due to the high incidence of CRC, there is an urgent need in the scientific research field to develop safe and effective drugs to treat CRC. Surgery, chemoradiotherapy, targeted, immune, and hormone therapy are the main methods for the treatment of CRC (3–7). For non-metastatic colorectal cancer, surgery alone or in combination with chemotherapy and radiotherapy is the mainstream approach. In contrast, metastatic colorectal cancer treatments encompass surgical resection, radiochemotherapy, neoadjuvant therapy, and immuno-targeted therapies (8). However, less than 20% of patients can achieve long-term recovery with surgery and chemotherapy (9). At the same time, due to the non-specific distribution of chemotherapy drugs, low efficacy, and high toxicity to the human body, conventional chemotherapy is no longer the best treatment (10). In order to solve the limitations of traditional treatment methods, researchers have invested great efforts to analyze the mechanisms of CRC and promote the development of new therapeutic drugs, which has promoted the exploration of nanomedicine and nanomedicine in CRC (11).

Nanomaterials have significant advantages in drug delivery, treatment and diagnosis of tumors (12, 13). At present, different drug carriers including nanoparticles, dendrimers, polymer micelles, liposomes, magnetic nanoparticles, and gold nanoparticles have been found (14). Various nanomaterials have their own advantages and disadvantages in cancer diagnostic and therapeutic applications. The wider application of nanomaterials in CRC therapy is to customize targeted drug delivery systems and advanced therapeutic modalities (15). Polymeric nanoparticles (PNPs) are one of the most widely used organic nanocarriers in colorectal cancer therapy, and the advantages of PNPs are their controlled drug release and physicochemical properties, long drug blood circulation time, good storage stability, and high drug loading (16). Polymeric micelles are also often used as organic nanocarriers for colon cancer therapy, and micelles with pH sensitivity have been shown to completely target colon cancer, thereby delivering controlled drugs to colon tissues with a release rate of more than 80% (17). Liposomal nanoparticles are widely used in drug delivery systems (DDS) due to their efficiency and multifunctionality, and their products for CRC treatment are still under clinical investigation (18). Some other inorganic nanocarriers, graphene-based nanomaterials, and composite nanomaterials have been analyzed for their value in the treatment of colorectal cancer (16). Through the association with nucleotides, genes, and many other bioactive substances, there is an opportunity to produce targeted nanomedicines with specificity, safety, and high efficiency (14). The main advantages of nanomedicine application in tumors are: (1) Active Targeting: Leverage specific recognition motifs to bind with tumor cells or the microenvironment for targeted therapy. (2) Passive Targeting: Utilize the enhanced permeability and retention (EPR) effect to boost drug delivery efficiency. (3) Indirect Targeting: Overcome limitations of tumor vascularization and microenvironment heterogeneity to selectively deliver drugs to tumors, offering new avenues for cancer treatment. (4) Reducing Side Effects: Employ passive and active targeting strategies to minimize drug distribution in healthy tissues, thereby reducing side effects on other organs. (5) Drug Release and Metabolism: Modify the release and metabolic properties of drugs to extend their circulation time in the body and enhance their stability and bioavailability. (6) Drug Synergy: Carry multiple drugs to achieve synergistic effects, improving outcomes against multidrug-resistant tumors and overcoming drug resistance (19). To sum up, the research and transformation of nanomedicine have broad prospects and application value in cancer treatment.

Due to the large amount of literature on nanomedicine and CRC, we used bibliometrics to summarize and analyze the field. Bibliometric analysis is a comprehensive scientific method to analyze articles in the field of study (20). We can visualize countries, institutions, and authors producing high-quality literature in the field and quickly identify research priorities and frontiers (21). In this article, we use software such as VOSviewer and Citespace to evaluate the latest advances in CRC and nanomedicine research. Our study helps scholars understand the frontiers and hot spots, and more effectively explore the relationship and transformation between CRC and nanomedicine.

## 2 Methods

### 2.1 Search strategy

**Figure 1:**
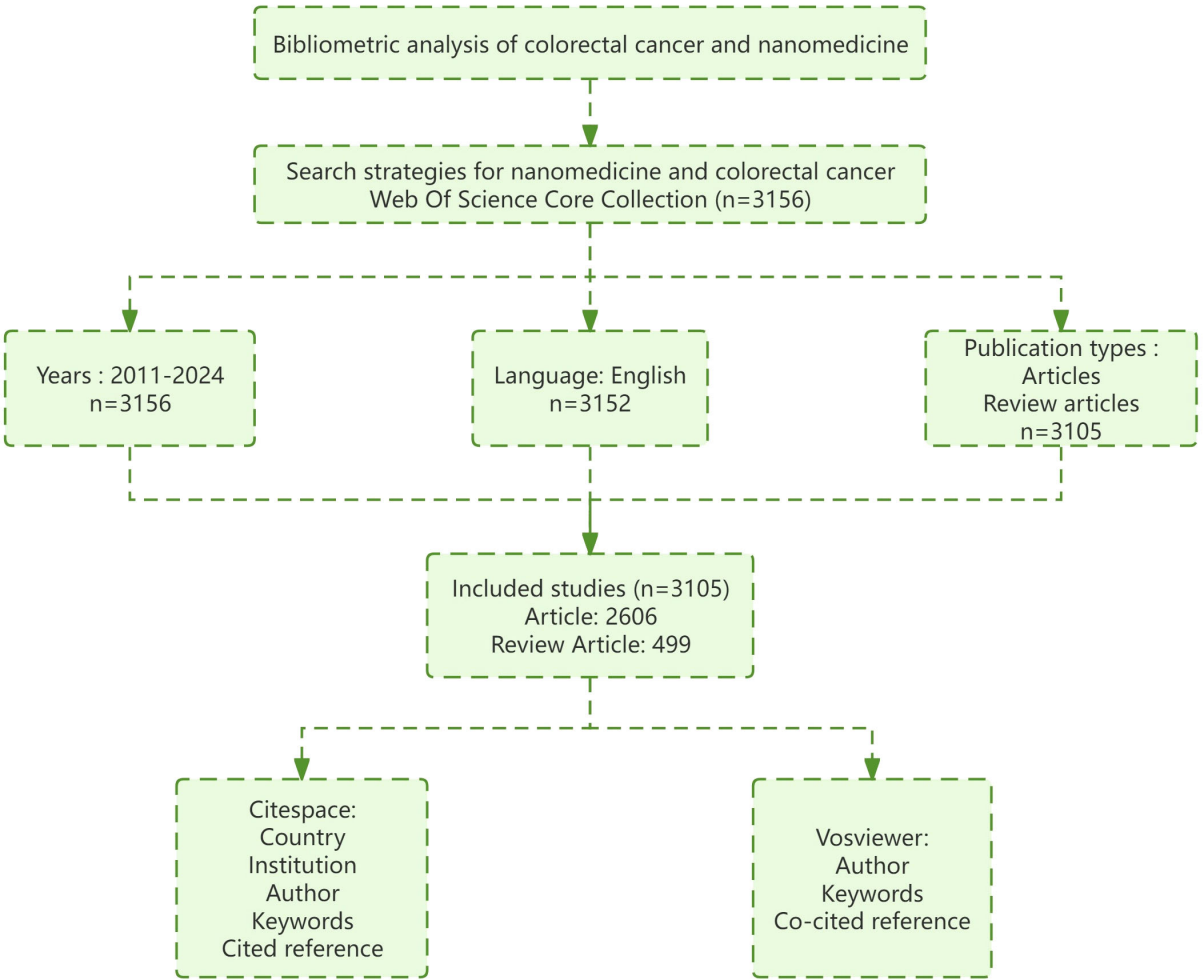
Document retrieval map.

The literature for this study comes from “English” papers published on the Web of Science Core Collection (WOSCC) between 2011 and 2024; “Article” or “Review article” is the type of literature needed for this study. We searched CRC and nanomedicine subject words and free words. The specific subject words and free words and their retrieval formula are as follows: TS = (nanomedicine) OR TS = (nanoparticle) OR TS = (nanocarrier) OR TS = (nanoparticles) OR TS = (nanocarriers) OR TS = (nanodrug) OR TS = (nanodrugs) OR TS = (nanotechnology) AND TS = (Colorectal Neoplasms) OR TS = (Colorectal Neoplasm) OR TS = (Neoplasm, Colorectal) OR TS = (Neoplasms, Colorectal) OR TS = (Colorectal Tumors) OR TS = (Colorectal Tumor) OR TS = (Tumor, Colorectal) OR TS = (Tumors, Colorectal) OR TS = (Colorectal Cancer) OR TS = (Cancer, Colorectal) OR TS = (Cancers, Colorectal) OR TS = (Colorectal Cancers) OR TS = (Colorectal Carcinoma) OR TS = (Carcinoma, Colorectal) OR TS = (Carcinomas, Colorectal) OR TS = (Colorectal Carcinomas) OR TS = (CRC). Finally, the data is exported in “plain text” format with “full records and citations”. The search was conducted independently by two researchers on March 12, 2024. **Figure 1** shows the flow of the research.

### 2.2 Software for analysis

This study used Microsoft Office Excel 2016, CiteSpace 6.3.R1, and Vosviewer 1.6.19 for analysis. We used Microsoft Office Excel 2016 to describe publishing trends and collate relevant data, and made a table CiteSpace 6.3.R1 for analyzing countries, institutions, authors, highly cited references and keywords, and drew a visual map. Vosviewer 1.6.19 was used to analyze and visualize authors, keywords, and co-cited references. A previously published article describes the setting of specific parameters of the CiteSpace (22).

## 3 Results

### 3.1 Publication trends

**Figure 2:**
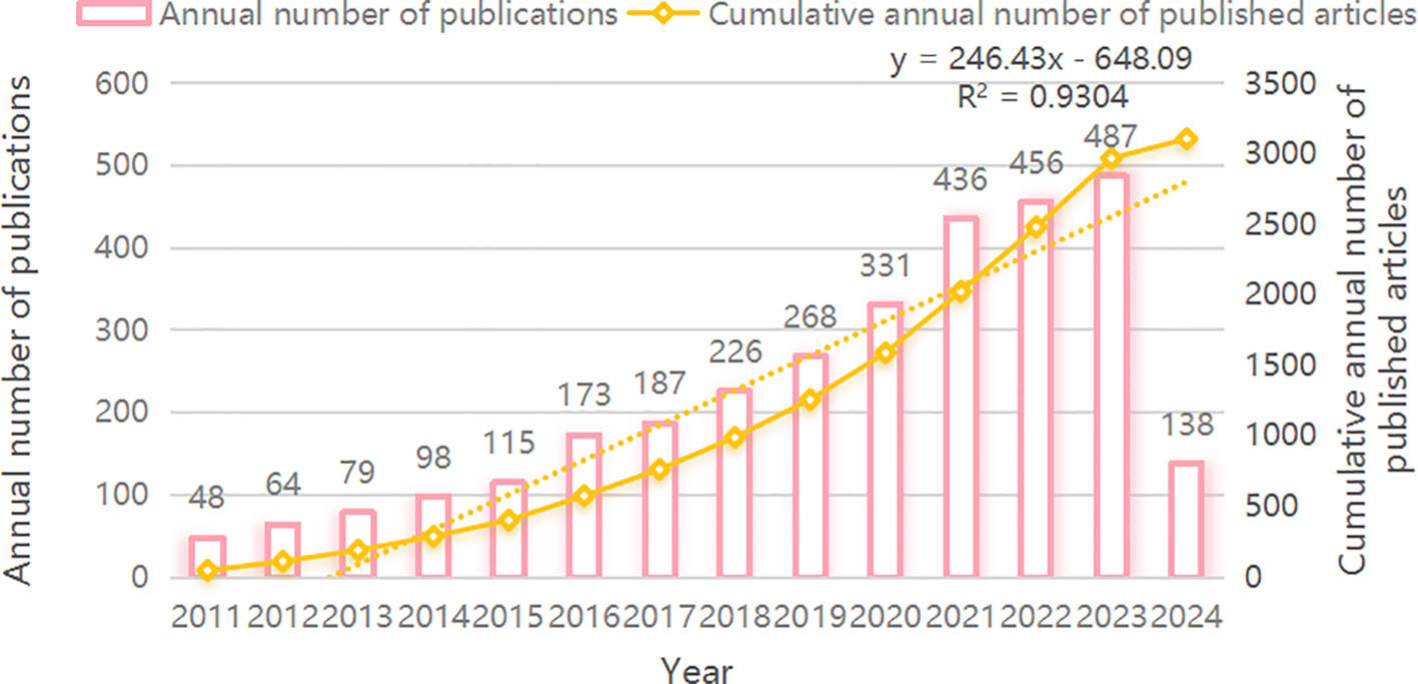
Published Trend Maps on CRC and nanomedicine.

A total of 3105 articles on CRC and nanomedicine were included in this study. As shown in **Figure 2**, from 2011 to 2022, the overall trend in the number of CRC and nanomedicine articles is upward, with a steady increase in the cumulative number. An exponential growth function was used to evaluate the link between the annual cumulative number of publications and the year, showing a strong correlation trend (R^2^ = 0.9304). This strong correlation shows that research into CRC and nanomedicine has come a long way. This represents a growing interest in CRC and nanomedicine research over the past 12 years.

### 3.2 Publications and collaborative networks

We surveyed publications and the centrality of countries, institutions, and authors in the field of CRC and nanomedicine. The larger the node size in the map is proportional to the number of papers by a single country, institution, or author. Purple rings appear outside nodes with centrality greater than 0.1. In addition, centrality is proportional to the collaboration of nodes.

### 3.3 Analysis of national publications and collaborations

**Figure 3:**
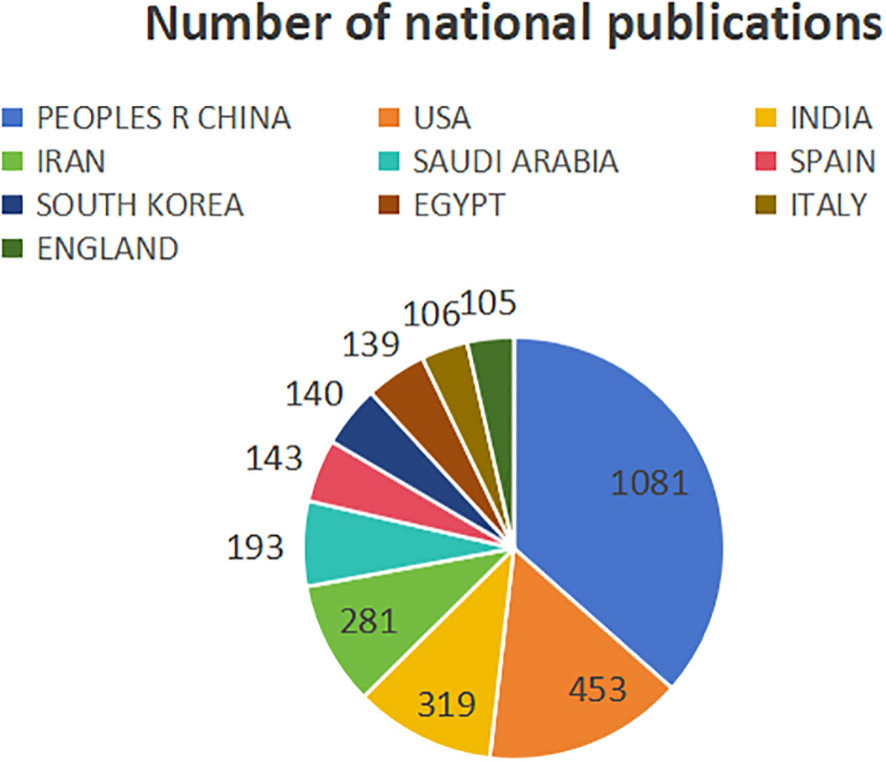
Number of publications by countries.

**Figure 4:**
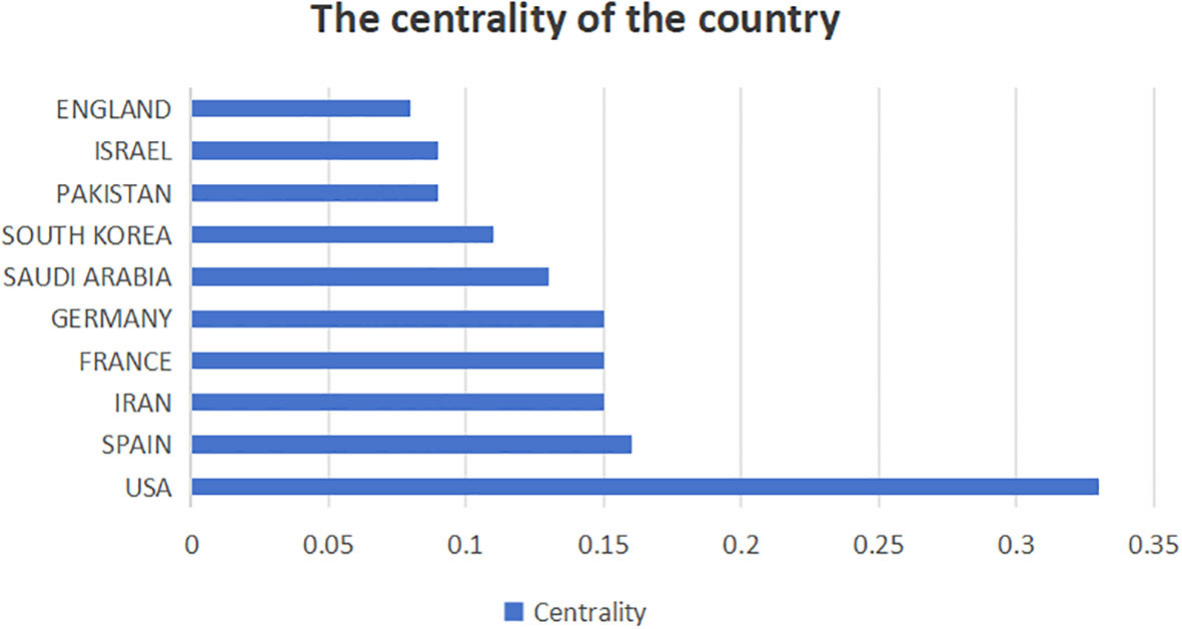
Intermediary centrality of countries.

**Figure 5:**
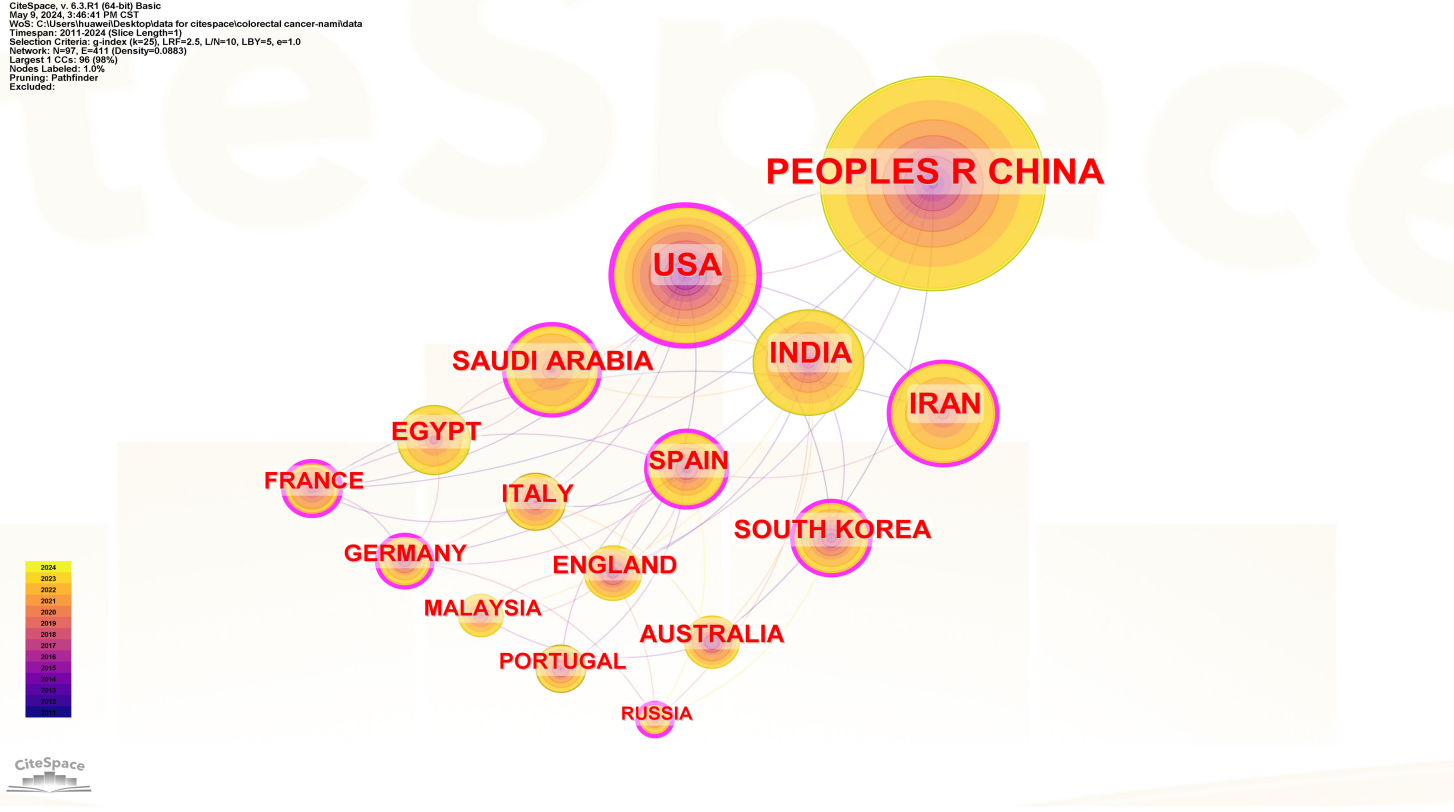
collaborative networks of countries.

This study analyzed the number of CRC and nanomedicine-related research publications (**Figure 3**), centrality (**Figure 4**), and collaborative networks (**Figure 5**) in different countries. **Figure 3** shows that China ranks first (1081 publications, 36.52%), followed by the United States (453 publications, 15.3%), INDIA (319 publications, 10.78%), IRAN (281 publications, 9.49%), and SAUDI ARABIA (193 publications, 6.52%). The remaining countries/regions did not reach 150 publications. **Figure 3B** shows that USA (0.33), SPAIN (0.16), IRAN (0.15), FRANCE (0.15), and GERMANY (0.15) are the five most central countries, with a strong correlation. Specific information on countries and the number of publications can be found in **Table 1**.

**Table 1:** Countries/regions, institutions, and authors ranked by publications and centrality.

Item	Rank	Name	Publications	Name	Centrality
Countries/Regions	1	PEOPLES R CHINA	1081(36.52%)	USA	0.33
	2	USA	453(15.3%)	SPAIN	0.16
	3	INDIA	319(10.78%)	IRAN	0.15
	4	IRAN	281(9.49%)	FRANCE	0.15
	5	SAUDI ARABIA	193(6.52%)	GERMANY	0.15
	6	SPAIN	143(4.83%)	SAUDI ARABIA	0.13
	7	SOUTH KOREA	140(4.73%)	SOUTH KOREA	0.11
	8	EGYPT	139(4.7%)	PAKISTAN	0.09
	9	ITALY	106(3.58%)	ISRAEL	0.09
	10	ENGLAND	105(3.55%)	ENGLAND	0.08
Institutions	1	Chinese Academy of Sciences	136(19.88%)	Shanghai Jiao Tong University	0.18
	2	Egyptian Knowledge Bank (EKB)	129(18.86%)	King Saud University	0.14
	3	Mashhad University Medical Science	68(9.94%)	Brigham & Women’s Hospital	0.14
	4	Zhejiang University	55(8.04%)	Centre National de la Recherche Scientifique (CNRS)	0.13
	5	King Saud University	54(7.89%)	Cairo University	0.13
	6	Sichuan University	51(7.64%)	Institut National de la Sante et de la Recherche Medicale (Inserm)	0.13
	7	Sun Yat Sen University	50(7.31%)	Chinese Academy of Sciences	0.12
	8	Fudan University	48(7.02%)	Imam Abdulrahman Bin Faisal University	0.12
	9	Jilin University	47(6.87%)	Tehran University of Medical Sciences	0.11
	10	Islamic Azad University	46(6.73%)	Egyptian Knowledge Bank (EKB)	0.10
Authors	1	Abnous, Khalil	12(21.43%)	Abnous, Khalil	0.00
	2	Alibolandi, Mona	11(19.64%)	Alibolandi, Mona	0.00
	3	Li, Wei	11(19.64%)	Li, Wei	0.00
	4	Huang, Leaf	11(19.64%)	Huang, Leaf	0.00
	5	Ramezani, Mohammad	11(19.64%)	Ramezani, Mohammad	0.00

### 3.4 Analysis of institutional publications and collaborations

**Figure 6:**
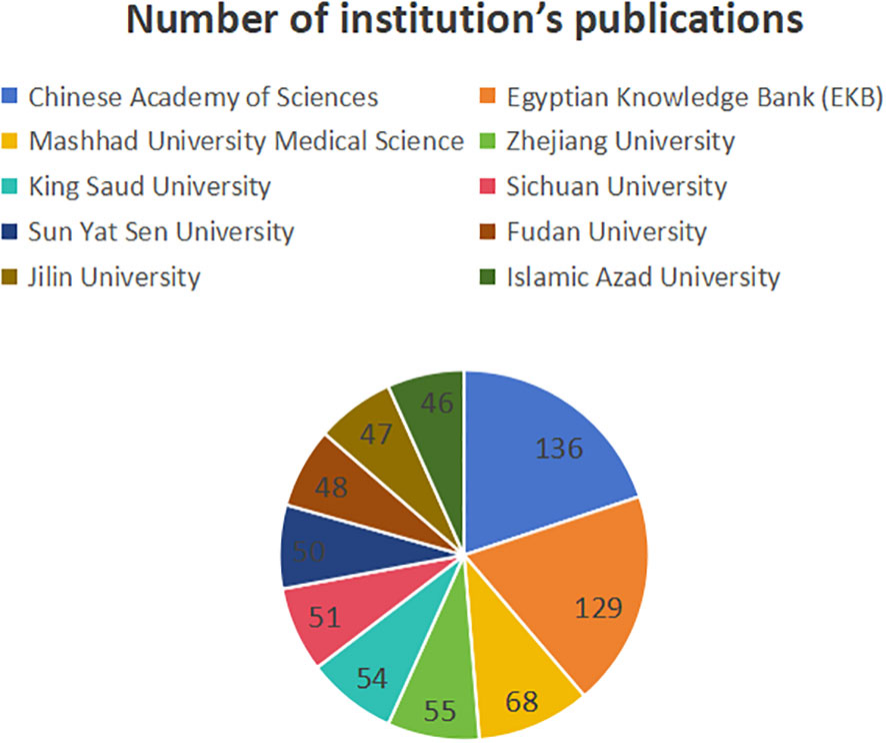
Number of publications by institution.

**Figure 7:**
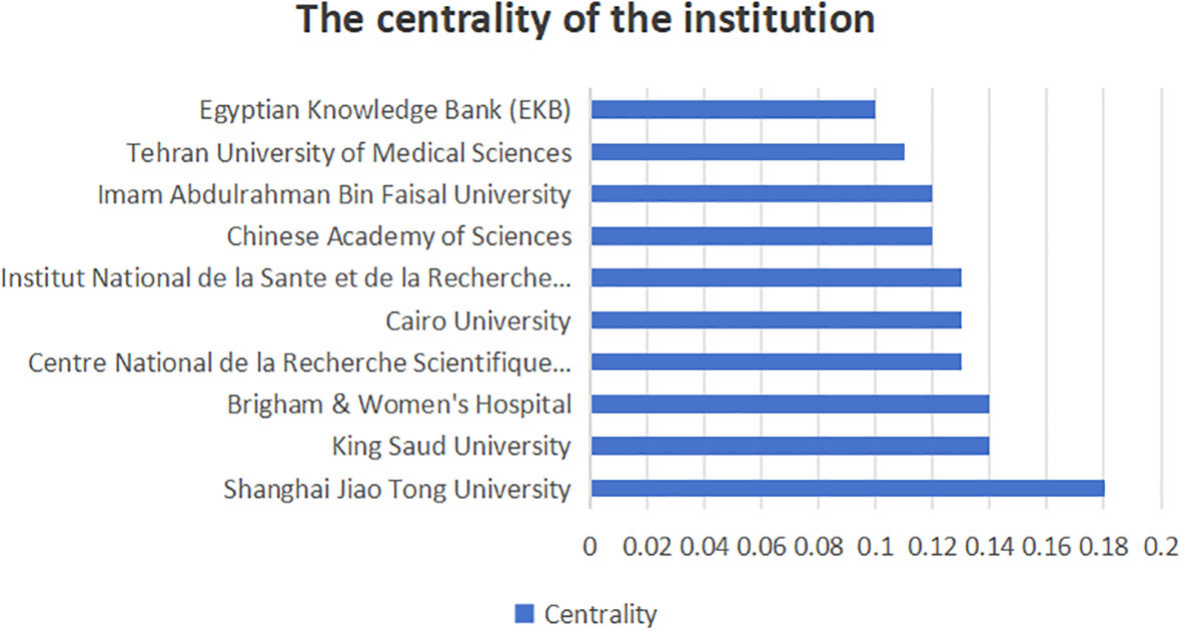
Intermediary centrality of institutions.

**Figure 8:**
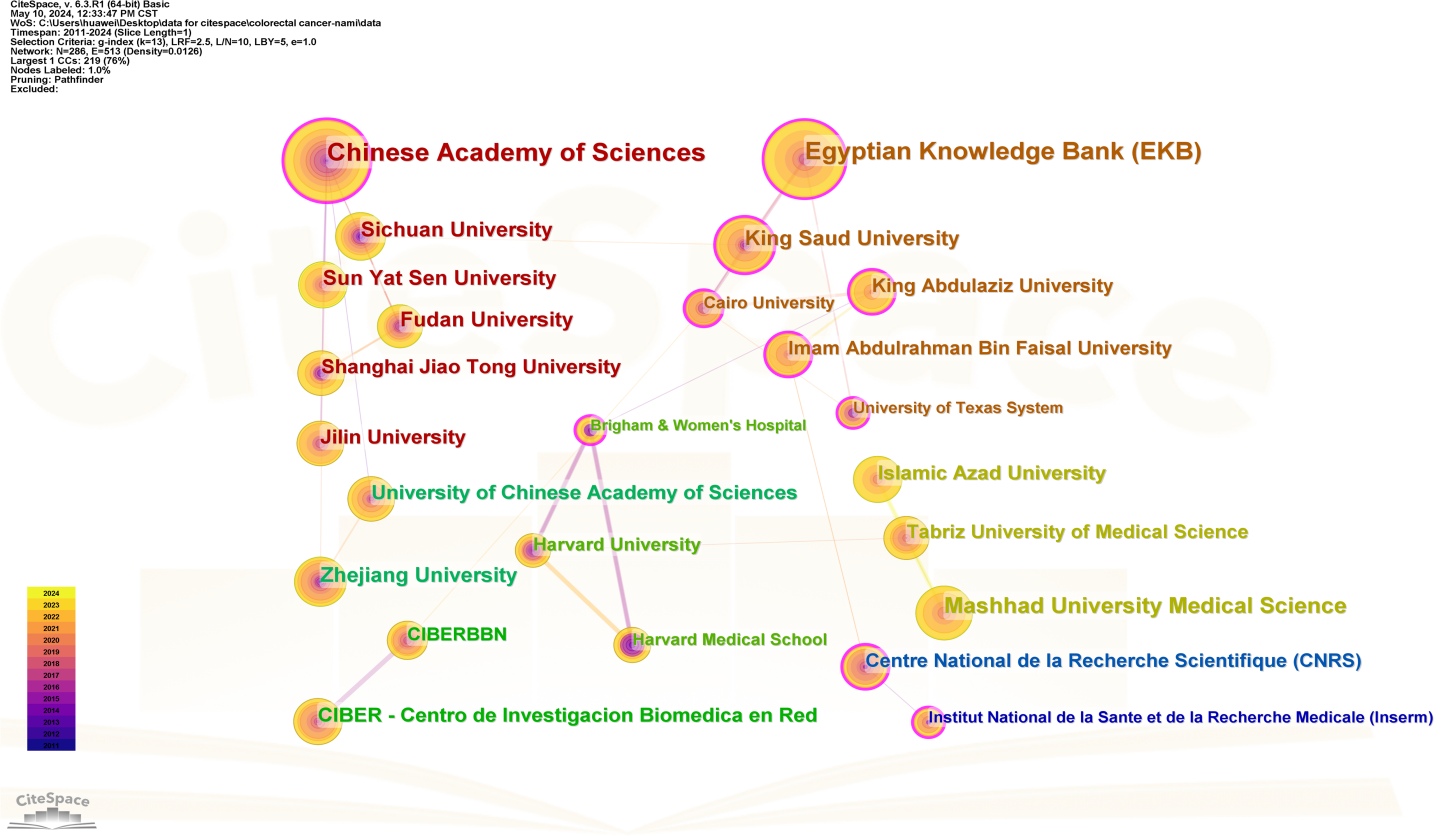
collaborative networks of institutions.

**Figure 6** shows the number of publications of the institution, while **Figure 7** shows the centrality of the institution and **Figure 8** shows the network of collaboration between the institutions. As you can see from this picture, the Chinese Academy of Sciences (136 publications, 19.88%), Egyptian Knowledge Bank (EKB) (129 publications, 18.86%), Mashhad University Medical Science (68 publications, 9.94%), Zhejiang Province University (55 publications, 8.04%) and King Saud University (54 publications, 7.89%) are the top five institutions. Shanghai Jiao Tong University (0.18), King Saud University (0.14), Brigham & Women’s Hospital (0.14), The centrality of institutions such as Centre National de la Recherche Scientifique (CNRS) (0.13) and Cairo University (0.13) is greater than 0.1, demonstrating the strong cooperation between institutions. Specific information on institutions and the number of published works can be found in **Table 1**.

### 3.5 Analysis of publications and cooperation among Authors

**Figure 9:**
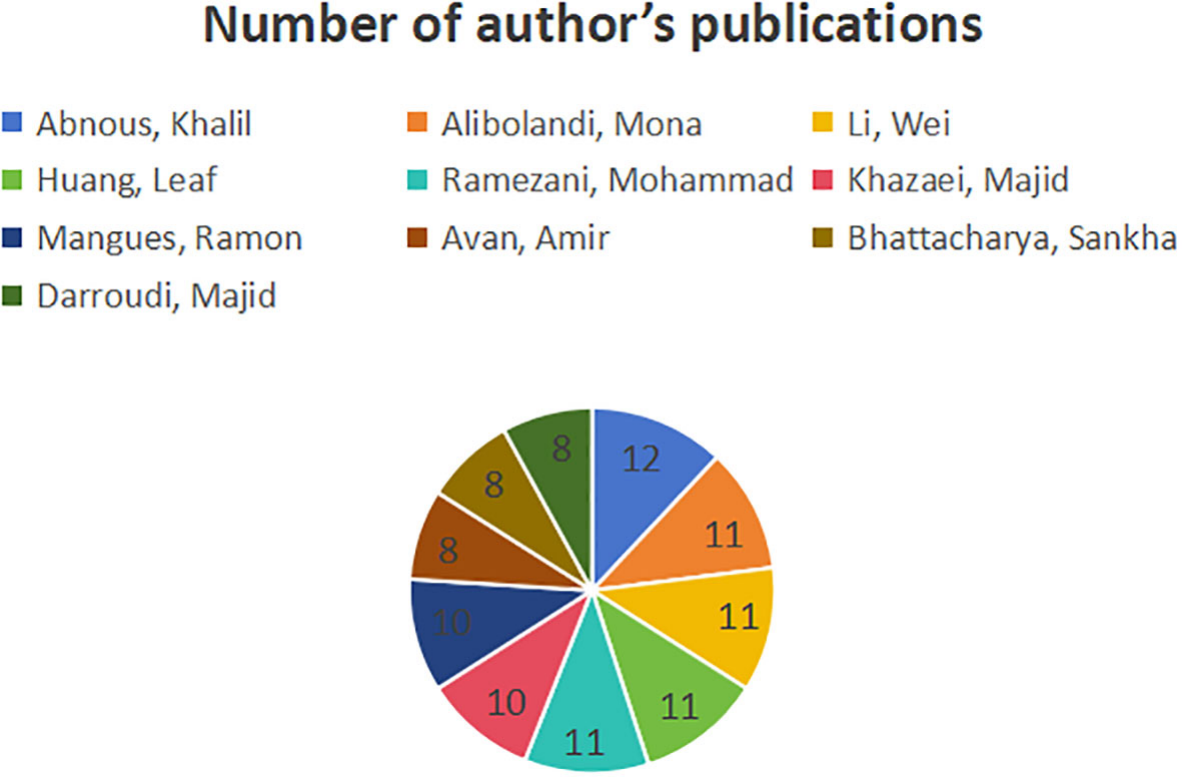
Number of publications by authors.

**Figure 10:**
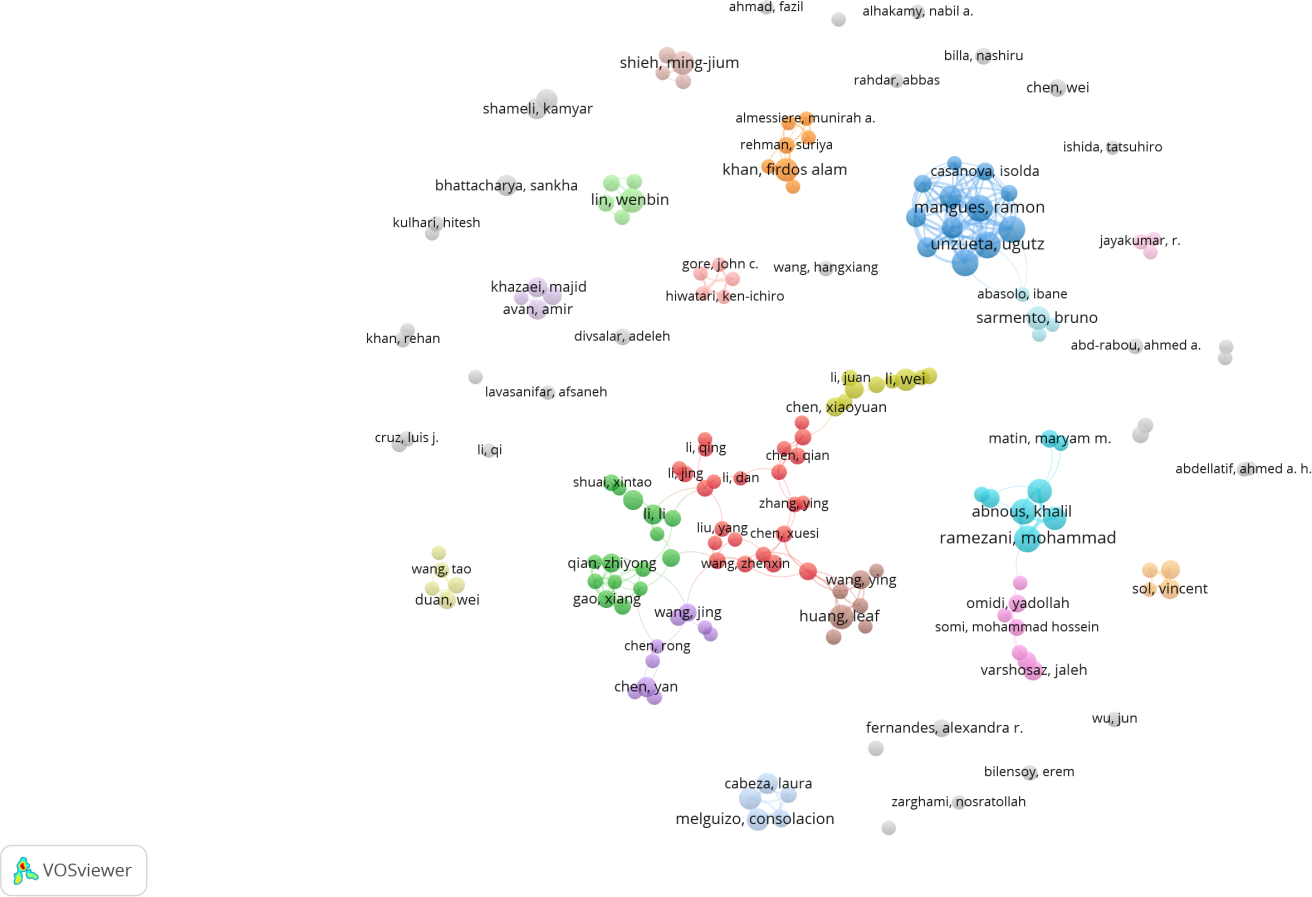
collaborative networks of authors.

**Figure 9** illustrates the publications by the authors, and **Figure 10** illustrates the cooperation network among authors. Abnous, Khalil (12 publications, 21.43%), Alibolandi, Mona (11 publications, 19.64%), Li, Wei (11 publications, 19.64%), Huang, Leaf (11 publications, 19.64%), Ramezani, Mohammad (11 publications, 19.64%) are the top five authors. The centrality of all authors is 0, indicating that a cooperative relationship does not exist among the authors, so the cooperation needs to be strengthened. Specific information can be found in **Table 1**.

### 3.6 Research hot spots and trend analysis

#### 3.6.1 Highly cited references and bursts

The increased number of citations can reflect the reliability and standardization of the research results of the article. According to the research topics of highly cited references, the research status and development direction of nanomedicine in colorectal cancer can be reflected to a certain extent. **Table 2** illustrates the top 10 highly cited references. The top cited article is named “Global Cancer Statistics 2020: GLOBOCAN Estimates of Incidence and Mortality Worldwide for 36 Cancers in 185 Countries “ (23). The study provides an up-to-date epidemiology of CRC. Colorectal cancer is the third most common and the second most deadly cancer in the world. Globalization and economic growth are likely to increase the global cancer burden, and efforts to build sustainable infrastructure to spread cancer prevention and care are critical to global cancer control. The article “Global patterns and trends in CRC incidence and mortality” in the top ten cited articles emphasizes that CRC cases and deaths continue to increase rapidly in low - and middle-income countries. Developed countries show a stable or declining trend (2). In addition to the relevant review of CRC and epidemiology, Shi J et al. mainly introduced the diagnosis and treatment of nanomedicine in tumors, and probes into the characteristics of nanotechnology application in oncology to provide reference for researchers (24).

**Table 2:** Top 10 highly cited references.

Item	Rank	Title	Journal	Citation
high-cited References	1	Global Cancer Statistics 2020: GLOBOCAN Estimates of Incidence and Mortality Worldwide for 36 Cancers in 185 Countries	CA Cancer J Clin (IF=254.7)	(112)
	2	Cancer Statistics	CA Cancer J Clin (IF=254.7)	(102)
	3	Colorectal cancer	Lancet (IF=168.9)	(73)
	4	Comprehensive review of targeted therapy for colorectal cancer	Signal Transduct Target Ther (IF=39.3)	(59)
	5	Global patterns and trends in colorectal cancer incidence and mortality	Gut (IF=24.5)	(58)
	6	Global colorectal cancer burden in 2020 and projections to 2040	Transl Oncol (IF=5.0)	(52)
	7	Epidemiology of colorectal cancer: incidence, mortality, survival, and risk factors	Prz Gastroenterol (IF=1.3)	(49)
	8	Colorectal cancer statistics, 2017	CA Cancer J Clin (IF=254.7)	(47)
	9	Cancer nanomedicine: progress, challenges and opportunities	Nat Rev Cancer (IF=78.5)	(46)
	10	Diagnosis and Treatment of Metastatic Colorectal Cancer: A Review	JAMA (IF=120.7)	(40)

**Figure 11:**
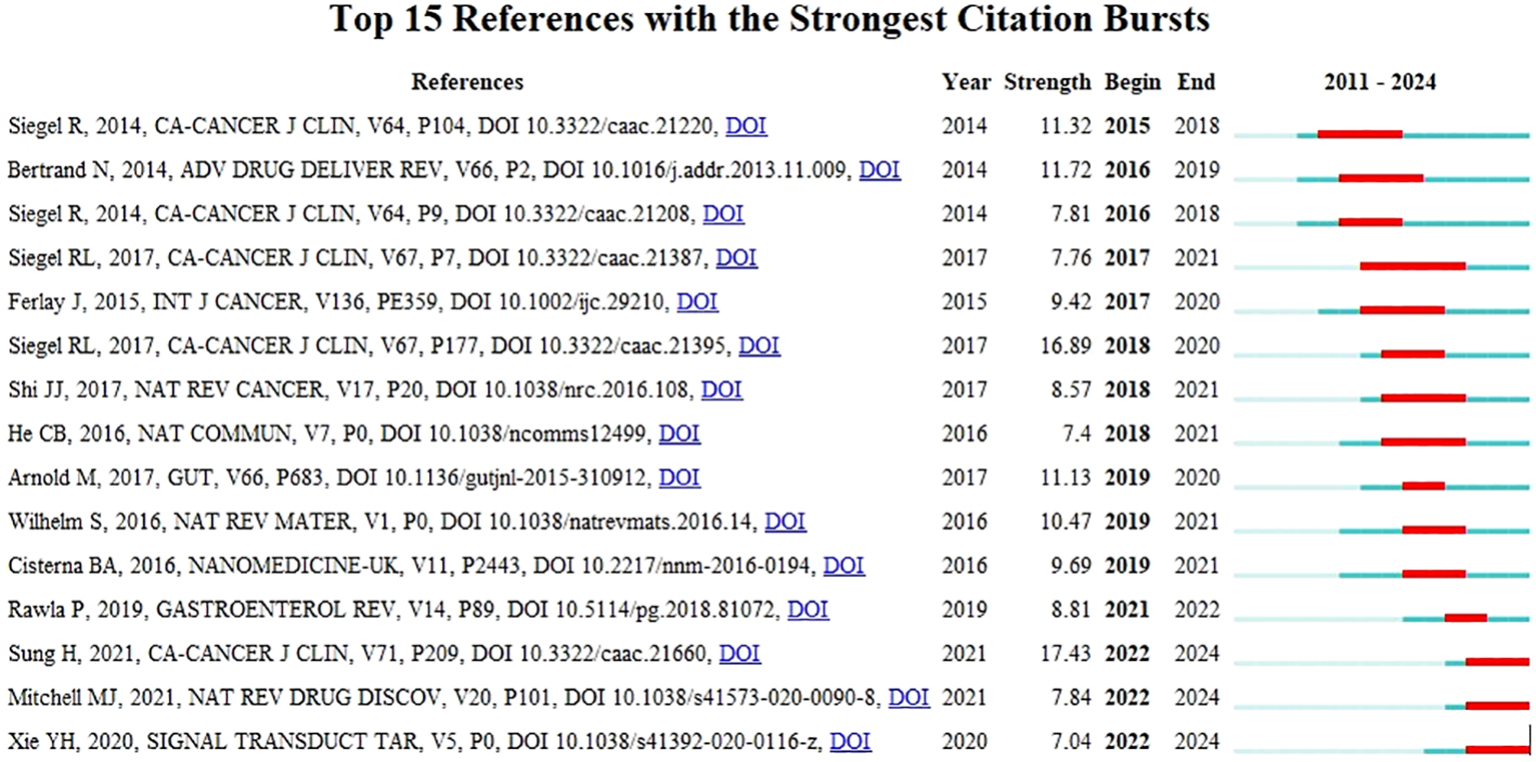
Highly cited references bursts.

References that are highly cited over a period of time are called references burst (25). As shown in **Figure 11**, a strong reference outbreak with a minimum duration of 1 year was found among the top 15 reference outbreaks. The recent explosion of citations has focused on the role of NPs in NDDS in tumors (18) and CRC-targeted drugs and their potential mechanisms (26). The co-cited references represent the development of nanomedicine in CRC.

#### 3.6.2 Highly co-cited references

**Figure 12:**
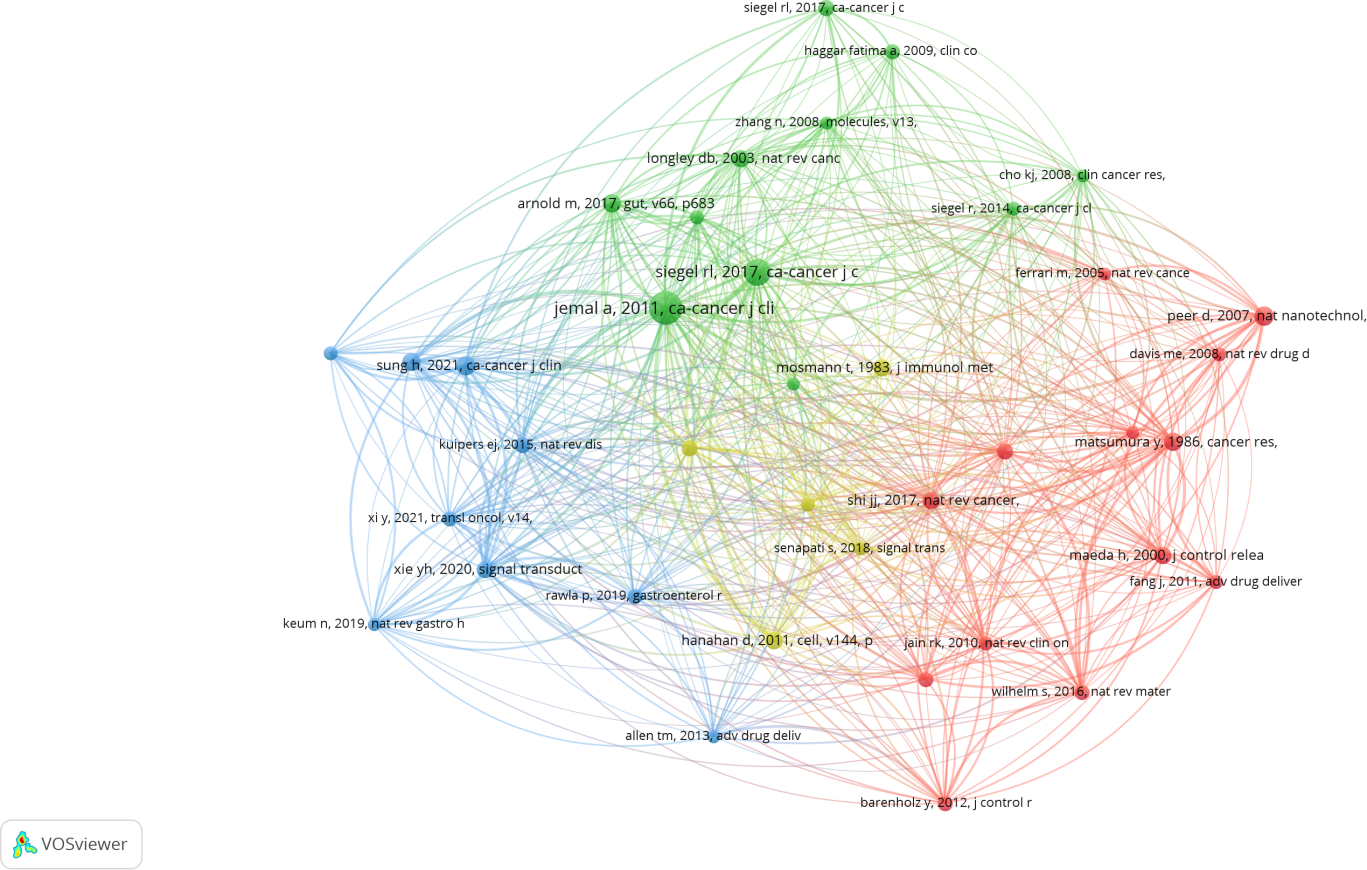
Cluster mapping of highly co-cited literature.

Co-cited references refer to the references that scholars cite together. Highly co-cited references is an important node in the academic cooperation network, which is conducive to understanding the dynamic development trend of nanomedicine. We use VOSviewer to visualize co-cited references. VOSviewer’s results show that the field collectively cited 146,575 references. When the number of citations is reduced to 36, 38 references remain. **Figure 12** illustrates that 38 references are divided into 4 clusters corresponding to 4 colors in the network diagram. The red cluster is mainly the basic research and related review of nanomedicine treatment of cancer. In 1986, Y Matsumura first found in the journal Cancer Research (IF=11.2) that solid tumors accumulate because the vascular system increases, macromolecules become more permeable, and blood vessels or lymphatic vessels rarely recover (27). That is, the enhanced permeability and retention (EPR) effects of lipids and macromolecular have laid the physiological basis for promoting the production of macromolecular-targeted anticancer drugs and nanomedicine (28). At the same time, the literature in the red cluster reviews the treatment of nanoparticles (29), nanocarriers( (30), nanotechnology (11), and nanomedicine (24, 31) in cancer, while noting that The FDA approved the first nanomedicine: Doxil^®^ (32). The green cluster is closely related to the epidemiology of CRC, that is, the global incidence and mortality of CRC. One of the literature published in Gut (IF=24.5) pointed out that the occurrence of CRC is related to the level of human development, and The number of cases and deaths from CRC continue to increase rapidly in many low - and middle-income countries. In highly developed countries, a stable or declining trend can be seen, which may be closely related to diet structure, and targeted preventive measures need to be taken (2). The blue cluster is related to reviews of CRC and its targeted therapies, including one review of the role of nanomedicine delivery systems in tumors (26). The literature in the yellow cluster describes tumor characteristics and tumor chemotherapy drug delivery vectors.

**Table 3** shows the top co-cited references, which mainly include the epidemiology, mechanism review of CRC, and the use of nanocarriers for cancer treatment. In the field of nanomedicine, the highly co-cited references mainly focus on review article, while the literature on clinical trials is relatively scarce. Future research directions should focus on carrying out more nanomedicine related clinical trials to promote scientific development and clinical applications in this field.

**Table 3:** Top 10 highly co-cited references.

Item	Rank	Title	Journal	Citation
co-cited references	1	Global cancer statistics	*CA Cancer J Clin* (IF=254.7)	(239)
	2	Cancer Statistics	*CA Cancer J Clin* (IF=254.7)	(157)
	3	Global Cancer Statistics 2020: GLOBOCAN Estimates of Incidence and Mortality Worldwide for 36 Cancers in 185 Countries	*CA Cancer J Clin* (IF=254.7)	(82)
	4	Nanocarriers as an emerging platform for cancer therapy	*Nat Nanotechnol* (IF=38.3)	(77)
	5	A new concept for macromolecular therapeutics in cancer chemotherapy: mechanism of tumoritropic accumulation of proteins and the antitumor agent smancs	*Cancer Res* (IF=11.2)	(76)
	6	Colorectal cancer	*Lancet* (IF=168.9)	(73)
	7	Global patterns and trends in colorectal cancer incidence and mortality	*Gut* (IF=24.5)	(71)
	8	Hallmarks of cancer: the next generation	*Cell* (IF=64.5)	(67)
	9	Tumor vascular permeability and the EPR effect in macromolecular therapeutics: a review	*J Control Release* (IF=10.8)	(67)
	10	Rapid colorimetric assay for cellular growth and survival: application to proliferation and cytotoxicity assays	*J Immunol Methods* (IF=2.2)	(65)

#### 3.6.3 Keyword co-occurrence, burst, and cluster

**Figure 13:**
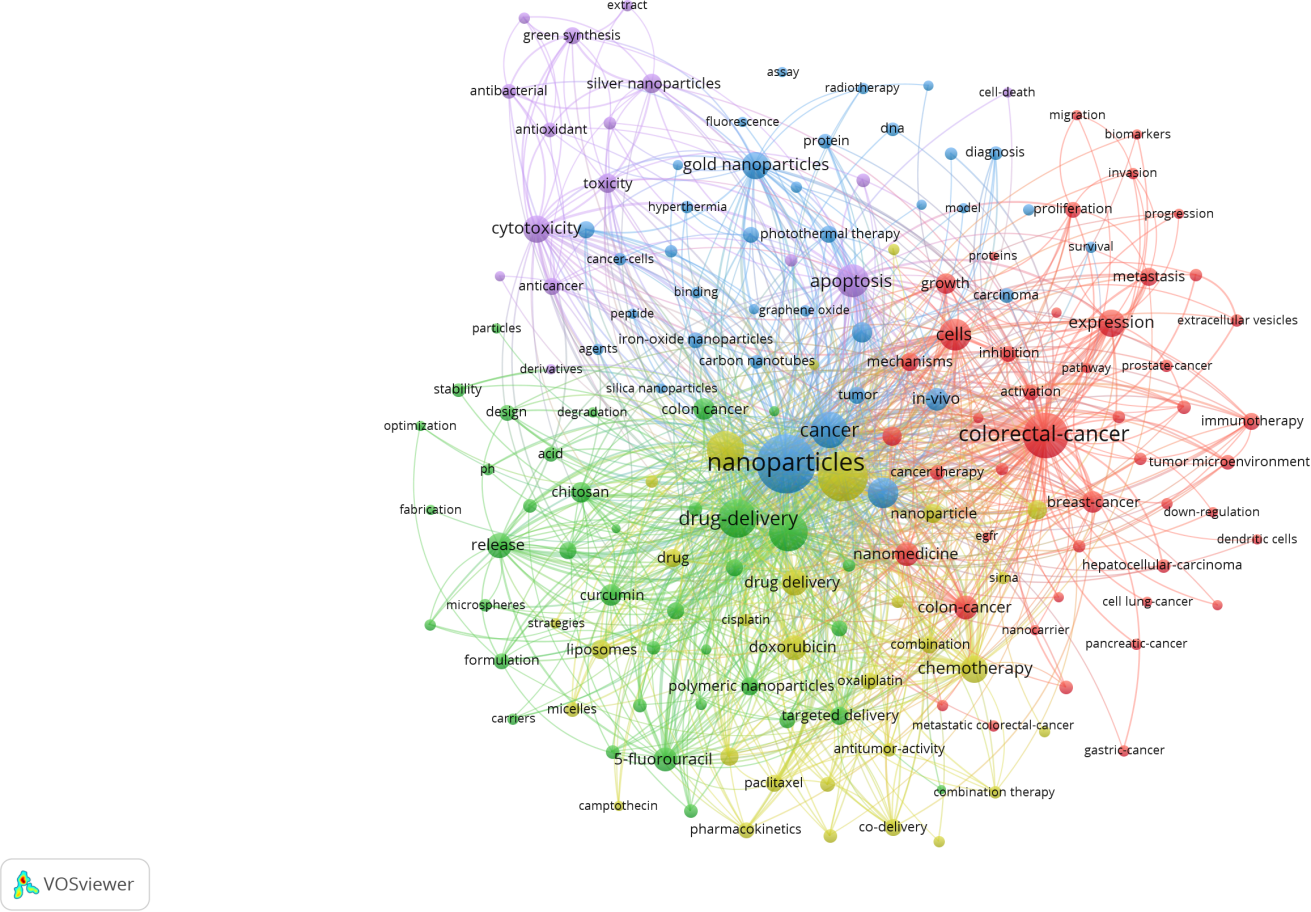
Keyword co-occurrence map of CRC and nanomedicine.

The co-occurrence of keywords is shown in **Figure 13**. Keywords with high frequency indicate the research hotspot of CRC and nanomedicine. The size of the nodes in the figure corresponds to their frequency of keyword occurrence. **Table 4** provides the keywords with the highest frequency in the keyword co-occurrence graph: Keywords co-occurrence graph of the highest frequency of keywords in the following: CRC, nanoparticles, drug delivery, *in vitro*, delivery, cells, and colon Cancer therapy, apoptosis, gold nanoparticles. Additionally, **Table 4** also provides the keywords with high centrality rankings in the keyword co-occurrence map: chitosan, drug delivery, cytotoxicity, activation, the design, CRC, colon cancer, apoptosis, cancer, and chemotherapy.

**Table 4:** Top 20 keywords in terms of frequency and centrality.

Rank	Keyword	Frequency	Keyword	Centrality
1	colorectal cancer	1323	chitosan	0.10
2	nanoparticles	791	drug delivery	0.07
3	drug delivery	599	cytotoxicity	0.07
4	*in vitro*	451	activation	0.07
5	delivery	411	design	0.07
6	cells	310	colorectal cancer	0.06
7	colon cancer	300	colon cancer	0.06
8	therapy	279	apoptosis	0.06
9	apoptosis	238	cancer	0.06
10	gold nanoparticles	234	chemotherapy	0.06
11	expression	231	oxidative stress	0.06
12	cancer	228	carcinoma	0.06
13	release	189	carcinoembryonic antigen	0.06
14	breast cancer	164	breast cancer	0.05
15	chemotherapy	160	doxorubicin	0.05
16	*in vivo*	152	acid	0.05
17	doxorubicin	140	drug delivery system	0.05
18	cytotoxicity	129	multidrug resistance	0.05
19	photodynamic therapy	128	nanomedicine	0.05
20	growth	127	carbon nanotubes	0.05

**Figure 14:**
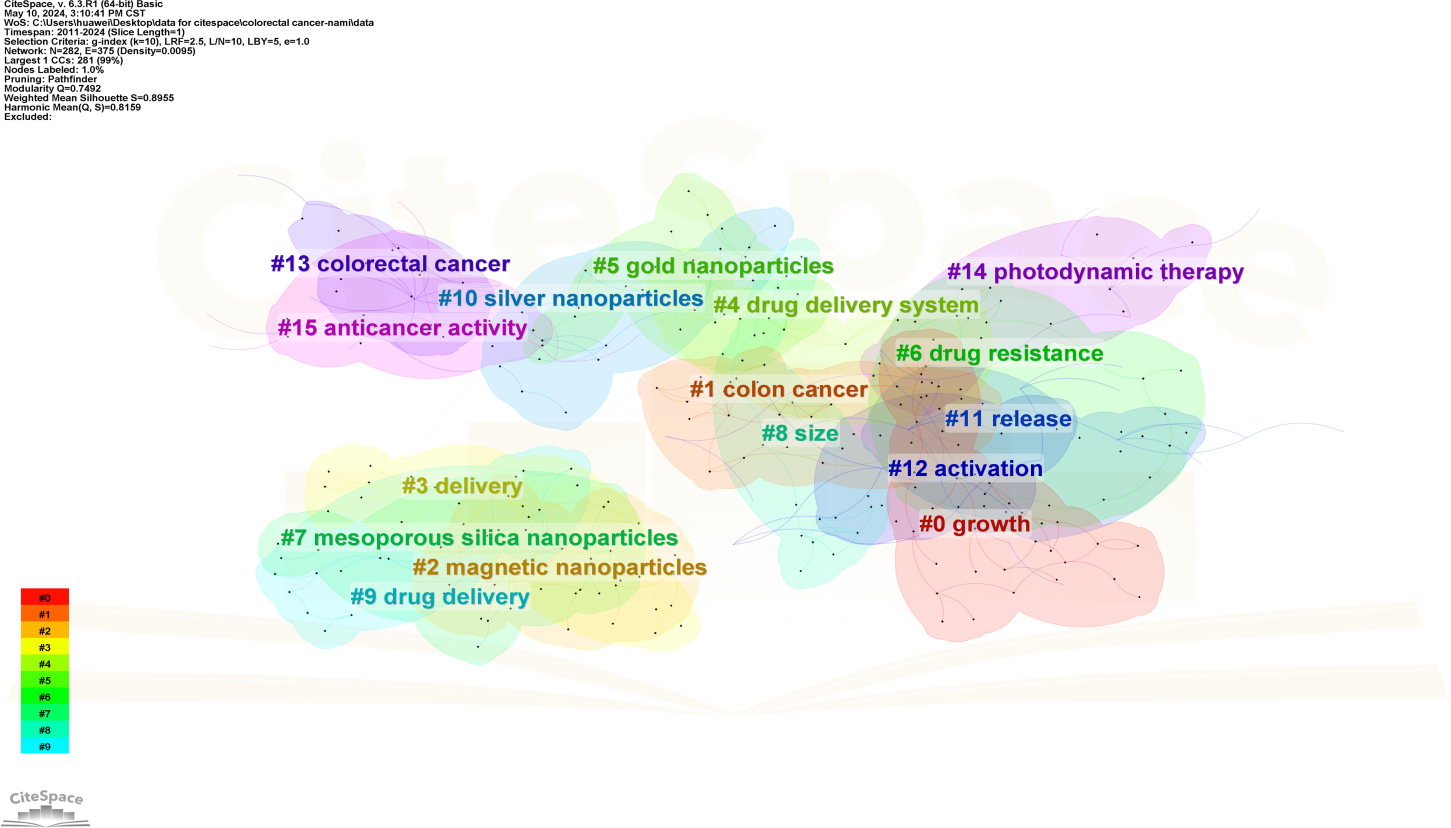
Keyword cluster map of CRC and nanomedicine.

In addition, CiteSpace will cluster closely related keywords. The higher the cluster rank, the more keywords contained in the cluster. Details about keyword clustering are shown in **Table 5**. A value greater than 0.5 in Silhouette represents the availability of keyword clustering. The study identified 16 clusters, #0 growth, #1 colon cancer, #2 magnetic nanoparticles, #3 delivery, #4 drug delivery system, #5 gold nanoparticles, #6 drug resistance, #7 mesoporous silica nanoparticles, #8 size, #9 drug delivery, #10 silver nanoparticles, #11 release, #12 activation, #13 CRC, #14 photodynamic therapy, # 15 anticancer activity (**Figure 14**).

**Table 5:** Keyword cluster analysis.

Cluster	Size	Sihouette	Mean year	Label (LLR)	Other keywords
0	29	0.928	2016	Growth	Oxidative stress; cancer cells; iron oxide nanoparticles; proliferation
1	23	0.857	2015	Colon cancer	Photothermal therapy; polymeric micelles; radiotherapy; immunogenic cell death
2	21	0.928	2013	Magnetic nanoparticles	*In vivo*; carcinoembryonic antigen; breast cancer; Cells
3	21	0.94	2014	Delivery	Expression; therapy; resistance; paclitaxel
4	19	0.89	2016	Drug delivery system	Cancer therapy; polymeric nanoparticles; inflammatory bowel disease; oral delivery
5	18	0.905	2013	Gold nanoparticles	Targeted therapy; apoptosis; carbon nanotubes; selenium nanoparticles
6	17	0.805	2017	Drug resistance	Plga nanoparticles; targeted drug delivery; folic acid; multidrug resistance
7	17	0.876	2017	Mesoporous silica nanoparticles	Plga nanoparticles; targeted drug delivery; folic acid; multidrug resistance
8	17	0.881	2015	Size	Gastrointestinal cancer; systems; silica nanoparticles; receptor
9	17	0.944	2016	Drug delivery	Extracellular vesicles; *in vitro*; docetaxel; self-assembly
10	16	0.745	2016	Silver nanoparticles	Extracellular vesicles; *in vitro*; docetaxel; self-assembly
11	15	0.898	2016	Release	Mucoadhesive; doxorubicin; design; solubility
12	14	0.862	2018	Activation	Hepatocellular carcinoma; signaling pathway; nf kappa b; drug delivery
13	13	0.944	2013	Colorectal cancer	Quantum dots; graphene oxide; circulating tumor cells; biosensor
14	13	0.965	2015	Photodynamic therapy	Microenvironment; photosensitizers; porphyrin; photosensitizer
15	11	0.973	2019	Anticancer activity	Toxicity; colorectal cancer; antimicrobial activity; gene expression

**Figure 15:**
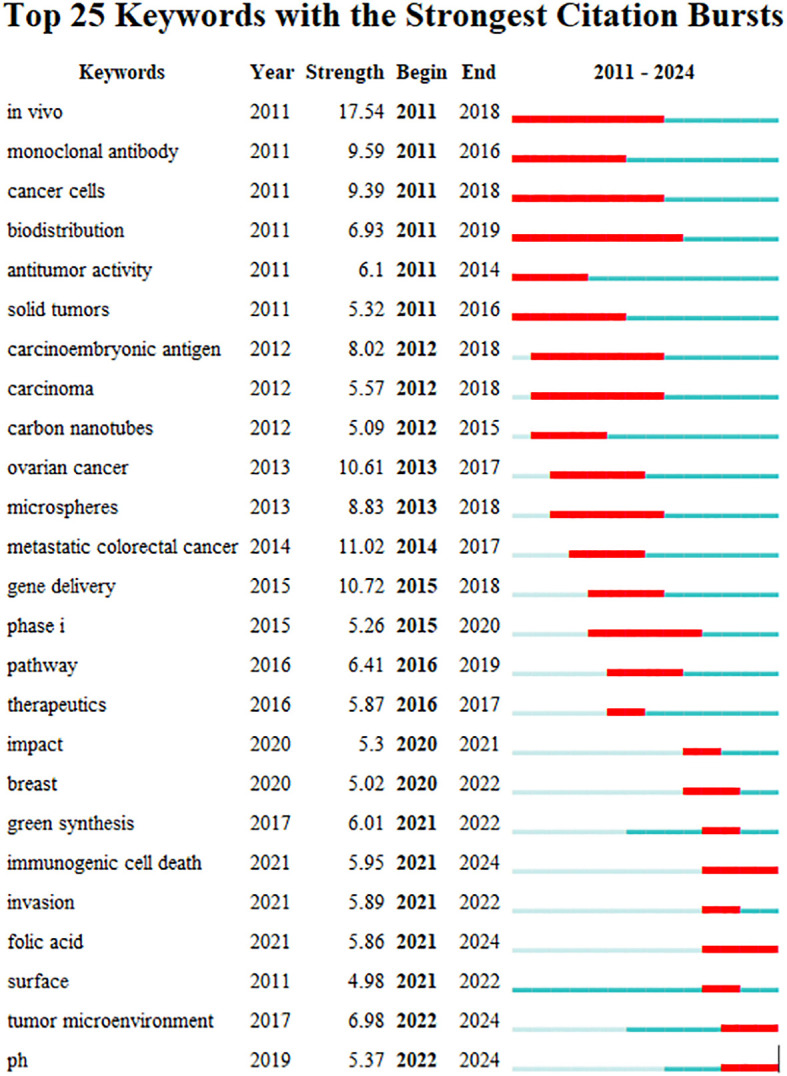
Keyword brusts map for CRC and nanomedicine.

Keyword burst is an explosive increase in a certain research over a period of time, which represents the future development trend of the research. **Figure 15** illustrates the top 25 keywords with the strongest burst intensity in this study. The red line in the figure represents the duration of the keyword burst. The five most powerful keywords include *in vivo*, metastatic CRC, gene delivery, ovarian cancer, and monoclonal antibody. Keyword outbreaks in the last 2 years include immunogenic cell death, folic acid, tumor microenvironment, pH.

## 4 Discussion

### 4.1 Publication trends and cooperation

Bibliometrics methods were used to analyze research progress in CRC and nanomedicine between 2011 and 2024. It is clear that the productivity of research in this field has increased as a result of worldwide attention. Over the past half-century, diets have changed dramatically, with low - and middle-income countries tending to consume more fat, sugar, and animal foods (33). At the same time, increased consumption of “junk” food, increased sitting, and decreased physical activity and standing have led to an increase in overweight and obesity and an increased incidence of CRC (34). Therefore, the prevention of colorectal cancer is very important. Policy and financial support for research institutions in many countries promote effective collaboration, thus contributing to the rapid growth of this research field.

### 4.2 National publishing trends and cooperation

Most of the top 5 countries/regions were from Asia, which is consistent with previous epidemiological surveys of CRC, so the attention of low - and middle-income countries on CRC is high and continues to rise. However, most of the top five centrality countries are still developed countries, indicating that developed countries have strong cooperation in this field and have significant influence. It is worth noting that China is the country with the most academic achievements in this field and is recognized as an emerging region in the field of nanomedicine, but the cooperation between China and other countries is low, and policies need to be introduced to strengthen the cooperation between relevant countries. For example, we can continue to promote and deepen the “Belt and Road” scientific and technological innovation cooperation, build an efficient and developed platform for scientific and technological cooperation, encourage scientific researchers to participate in international academic exchanges, and reform and innovate the mechanism for funding international academic research. The United States, as the most cooperative power, has a broader influence and can lead the continued development of this field.

### 4.3 Institutional publishing trends and cooperation

Depending on the number of publications of the institution, the top 10 organizations are all from Asia, with the largest number coming from China. Chinese Academy of Sciences, Egyptian Knowledge Bank (EKB), Mashhad University Medical Science, Zhejiang University, and King Saud University account for the majority of published works, which are major drivers of nanomedicine and CRC research. Meanwhile, Shanghai Jiao Tong University, King Saud University, Brigham & Women’s Hospital, Centre National de la Recherche Scientifique (CNRS), and Cairo University have the highest centrality and have close links and cooperation with other institutions. This emphasizes the importance of seeking partnerships to enhance research competitiveness within economic or resource constraints.

### 4.4 Author’s publishing trends and cooperation

Abnous, Khalil, Alibolandi, Mona, Li, Wei, Huang, Leaf, Ramezani, Mohammad are the most influential scholars in this field, and their research content represents the frontier and direction of research. However, there is no collaborative relationship between the authors, and it is necessary for states and institutions to take steps to provide resources and platforms to strengthen collaboration among authors and thus move the field forward. For example, the establishment of international scientific and technological cooperation agencies, the building of platforms for scientific and technological exchanges as well as the organization of national and international academic conferences to enhance academic exchanges among scholars, and the provision of research funding for multi-author collaborative projects.

### 4.5 Research basics and hot spots

The research hotspots and frontiers of CRC and nanomedicine can be demonstrated through bibliometric analysis. Highly co-cited references, highly cited references, and keyword analysis show that nanoparticles (NPs) (gold nanoparticles (AuNPs), silver nanoparticles (AgNPs), mesoporous silica nanoparticles (MSNs)), nanomedical drug delivery system (NDDS) are the focus and hot spot in this field. At the same time, we found from keyword clustering that photodynamic therapy (PDT) is a cutting-edge topic in this field and seems to be an interesting topic. Keywords burst identified immunogenic cell death (ICD), tumor microenvironment (TEM), folic acid, and pH as the forefront and development trend of this research.

#### 4.5.1 The role of NPs in CRC

Nanotechnology is a booming field, especially with the emergence of NPs as a coating agent for nanomedical drug delivery and pathological site imaging (35). Particles between 1 and 100nm with special surface properties are called NPs (36). NPs achieve targeted delivery, increase drug stability, and reduce toxicity to other tissues and organs (37). Targeted NPs are NPs that can specifically recognize cells. Because tumors express many biomarkers, these biomarkers can be used as targeted NPs for drug delivery. For example, Graf et al. described how targeting NPs could be a powerful approach to cancer treatment by binding to integrins that are involved in tumor angiogenesis (38). In addition, NPs help in the early diagnosis of CRC. He et al. described that NPs composed of ferric oxide magnetite and gold can perform dual-mode imaging in nude mice (SW620) carrying CRC (39). Metal NPs are booming in the biomedical field. AuNPs are targeted delivery vectors of molecular organisms (40). AuNPs are an effective drug that inhibits tumor growth in colorectal cancer by decompressing blood vessels (41). AgNPs are widely used as antibacterial and anti-inflammatory agents, drug delivery carriers, imaging probes, and optoelectronic platforms (42). AgNPs can penetrate the cell barrier to enter cells, trigger tumor cell apoptosis and inhibit tumor cell proliferation (43). MSNs are biocompatible and thermologically stable vectors that can deliver miRNA mimics or anti-miRNAs to target cells and inhibit the growth of cancer cells. MSNs-anti-miR-155@PDA-Apt is a promising NP for the treatment of CRC (44).

#### 4.5.2 NDDS in CRC

NDDS in the field of colorectal cancer is an emerging field with a promising future (45). NDDS has made substantial progress over the past few decades (46). These systems can prevent targeted drugs from being degraded in circulation, reduce drug toxicity, and improve treatment outcomes (47). NDDS can significantly affect the treatment outcome of CRC. NDDS can add targeting sites such as antibodies or ligands on its surface to improve the targeting binding ability and avoid off-target effects (48). NDDS can precisely target the components of TME and regulate tumor progression through TME. Compared with previous treatment methods, NDDS offers several advantages, including enhancing drug solubility and bioavailability, precisely targeting cancer cells, controlling and sustaining drug release, reducing side effects and toxicity, and overcoming drug resistance (49). The combination of bacteria with NDDS can extend the therapeutic advantages of solid tumors, including colorectal cancer (50). Targeted nanomedicine is delivered to the colon, and nanoparticles accumulate in the colorectal area to achieve local treatment, improve the treatment effect, and reduce systemic toxicity. However, factors such as sudden release of the drug, degradation of enzymes and acids in the stomach, and changes in pH may prevent NDDS from being successfully delivered to the colon, so advances in NDDS have the potential to overcome these challenges and thus provide effective drug delivery for CRC (48).

#### 4.5.3 Opportunities and challenges of PDT in CRC and nanomedicine

PDT is a minimally invasive method that has captured significant attention as an emerging tool in cancer treatment (51). In PDT, photosensitizers (PSs) generate reactive oxygen species (ROS) upon activation by light of specific wavelengths. These reactive oxygen species induce cytotoxic effects, leading to the death of tumor cells (52). NDDS can be used in the treatment of colorectal cancer by PDT. However, due to factors such as limited skin light penetration that limit the efficacy of PDT in CRC, the application of NPs in PDT can increase the efficacy (53). NPs typically exhibit relatively large surface area increases that interact with photoactivable photosensitizer(PS) surfaces to enhance uptake by cancer cells (54). NPs protect PS from immune system barriers, prolong the circulating life of PS, and avoid side effects (54). In addition, smaller NPs can increase the transport of PS to target cells through EPR effects (55). NP carrier platform is a favorable platform for PS delivery because of its unique physical, chemical, and biological characteristics (56).

#### 4.5.4 Nanomedicine mediates immunotherapy of CRC through ICD

Nano-drugs that target CRC cells often present tumor-associated antigen (TAA) in a way that triggers ICDs (56). Macrophages and dendritic cells (DC) capture these immunogenic factors and present them to CD8 cytotoxic T cells (57). The antigen-specific immune response of this process can directly and effectively kill cancer cells, and enhance the anti-tumor effect by releasing TAA (58). The feasibility of this principle has been verified by monoclonal antibody (MAB). Icd-inducing NPs increase the infiltration of immune effector cells targeting tumor cells (59). Radiotherapy and PDT can lead to the death of tumor cells and induce ICD. Thus, these treatment modalities actively integrate NPs with ICD-inducing properties. Nanomedicine is a promising approach to combat the immune evasion of cancer cells and inhibit cancer metastasis.

#### 4.5.5 Challenges and opportunities of the TME for nanomedicine treatment in CRC

TME presents challenges for the treatment of nanoparticles in CRC, including irregular tumor vascular networks, elevated tumor interstitial fluid pressure, and the presence of extracellular matrix (ECM) (60). Elevated tumor interstitial fluid pressure (TIFP) results from the rapid proliferation of tumor cells within confined spaces, the release of a plethora of angiogenic factors, vascular leakage within the tumor microenvironment, lymphatic vessel maldevelopment, and stromal fibrosis. Specifically, this phenomenon is attributed to the lack of normal lymphatic vessels within the tumor, coupled with the overexpression of lymphangiogenic factors, which promotes the formation of immature lymphatic vessels. The combined effects of these factors result in impaired drainage capacity (61). Additionally, areas of hypoxia arise within the rapidly growing tumor vasculature due to insufficient blood supply, further stimulating the production of various angiogenic factors (62). This, in turn, leads to the development of a vascular system characterized by low stability, loss of cellular continuity, and heightened endothelial activation, contributing to vascular permeability in the tumor microenvironment. Moreover, this process stimulates the tumor ECM, exhibiting signs of fibroblast differentiation into smooth muscle cells, thereby rendering the ECM denser and more rigid (63). All of the aforementioned factors impede the convective transport of oxygen, nutrients, and chemotherapeutic agents within the tumor, leading to inefficient drug penetration and distribution, which subsequently results in slower tumor proliferation and the development of resistance to anticancer therapies (64). The ECM is primarily composed of glycoproteins, proteoglycans, glycosaminoglycans, and collagen, all of which exert varying degrees of influence on tissue transport capabilities. Research has demonstrated that a dense tumor ECM affects the transport of macromolecules such as antibodies (65). To address the hypoxic environment caused by TIFP, current nanotechnology is mitigating hypoxia by developing classic oxygen carriers with nan formulations and novel artificial oxygen modulators (66). The solution is to improve tumor blood perfusion by restoring the tumor vascular system, promote nanomaterial extravasation by enhancing vascular permeability, and enhance drug transport by cell reprogramming (67). Compared with traditional high-concentration administration, stimulating the proliferation of immune cells can produce a long-term effective anticancer effect (68). Therefore, it is necessary to design non-tumor targeting NPs so that they can deliver immune stimulants leading to the generation of anti-tumor responses without entering the tumor interior.

#### 4.5.6 Folic acid improves drug delivery of CRC by modifying NPs

Folic acid, one of the most widely utilized molecules in current targeted drug delivery systems, is recognized by folate receptors on the surface of cancer cells, leading to the formation of folate-receptor complexes (69). These complexes subsequently generate endocytic vesicles that facilitate the internalization of folic acid into cancer cells (70). Notably, folate is overexpressed on the surface of approximately 40% of solid tumors, while its expression in healthy tissues is negligible (71). Therefore, leveraging the differential expression of folate in normal and tumor cells allows for its conjugation to the surface of NPs as a targeting moiety. This modification enables selective delivery of drug-loaded systems to tumor cells, as both folic acid and folate-conjugated compounds exhibit a degree of cancer selectivity, thereby enhancing the efficacy of targeted anticancer therapy (72). The studies have demonstrated that folate receptor (FR) is overexpressed in colorectal cancer, and the utilization of folate-loaded nanomaterials enables precise tumor localization by targeting folate-FR. Folate-modified nanoparticles can serve as effective drug delivery vehicles for achieving quantitative control of drugs and ensuring sustained drug release during the delivery process in colorectal cancer (73). Targeted delivery through folic acid-modified Nps maintains high blood concentrations and minimizes the toxic effects of the drug on normal tissue (74). Studies have demonstrated that folic acid-modified targeted NPs can enhance targeting specificity and drug retention duration through endocytosis. One article reported that folic acid-modified nanoparticles showed reduced toxicity and enhanced targeting in cells (75). Another article found that folic acid-modified paclitaxel lipid polymer NPs had better anticancer effects than unmodified NPs due to endocytosis (76).

#### 4.5.7 The role of pH-sensitive nanotechnology in CRC

The tumor acidic microenvironment (pH < 6.8) is a prominent characteristic within the human body. pH-responsive nanoparticles can facilitate targeted drug delivery to tumors. Furthermore, pH-responsive nanomaterials can effectively enhance the drug release profile, while the acidic tumor stroma environment can augment the uptake rate of nanocells. In colorectal cancer, pH-responsive nanomaterials have the potential to specifically modulate gut microbiota and restore balance to an imbalanced gut microbiota (77, 78). Due to the presence of the Warburg effect in tumor cells, which is characterized by elevated glycolytic rates in normoxic conditions, there is an increased production and secretion of lactate. Additionally, tumor cells generate substantial amounts of carbon dioxide through oxidative metabolism. Consequently, one of the defining characteristics of the tumor microenvironment is acidosis, a phenomenon that also applies to CRC (79). Therefore, pH-sensitive NPs exhibit pH responsiveness, which enables them to remain stable in systemic circulation, thereby reducing metabolic degradation and minimizing toxic side effects (80). In specific environments, such as the acidic microenvironment of CRC, these nanoparticles undergo degradation or conformational changes, triggering the release of the therapeutic agent from the NPs and facilitating targeted treatment (81). Currently, there are two primary mechanisms for drug release from pH-sensitive NPs: (1) Protonation and charge reversal triggered by acidic stimuli, which leads to structural disruption of the nanocarrier, thereby facilitating the specific release of the therapeutic agent. (2) Incorporation of pH-sensitive dynamic chemical bonds within the NPs, which undergo cleavage in acidic environments, resulting in conformational changes of the nanocarrier that regulate drug release. This process ensures effective delivery of the therapeutic agent to tumor tissues (82–84). Targeting NP polymers with similar pH values to the colorectal is a reliable technique. There are many advantages to using these polymers, such as low pH effects and enhanced absorption and bioavailability of acidic and insoluble drugs (85). Multiple studies have confirmed that the development of pH -sensitive NPs contributes to the treatment of CRC (86–88).

#### 4.5.8 biomimetic cell membrane improves drug delivery of CRC by modifying NPs

The cell membrane-coated nanoparticles utilize the cell membrane as a carrier to enhance blood circulation, achieve immune evasion, and facilitate long-term circulation and targeted delivery of intranuclear nanoparticles *in vivo*. They can be categorized into various types including erythrocyte membrane, macrophage membrane, NK cell membrane, DC membrane, cancer cell membrane, platelet membrane, and mesenchymal stem cell membrane (89). The cell membrane-coated nanomaterials (CMCNPs) in colorectal cancer can mimic the characteristics of source cells, enhance tumor targeting ability, evade ER clearance, prolong circulation time in the bloodstream, and achieve immune evasion thereby improving drug delivery accuracy and efficiency (90). Moreover, by integrating various therapeutic modalities such as photodynamic therapy, photothermal therapy, and immunotherapy cell membrane-coated nanomaterials offer novel prospects for cancer treatment (91). Studies have demonstrated that employing HT-29 tumor cell membrane (TCM) as the outer shell with paclitaxel (PTX)-loaded gelatin nanogel (GN) as the core biomimetic nanodrug delivery system (TCM/GN/PTX) effectively reduces PTX release and enables selective targeting towards tumor cells of the same type, thereby enhancing drug accumulation at the tumor site and significantly suppressing tumor growth (92). Currently, researchers are also combining different types of membranes to create hybrid nanoparticles for drug targeting and improved drug delivery. Et al., discovered that a hybrid nanoparticle coated with a MSIRPα-overexpressing cell membrane (CM-MSIRPα), denoted as MPB-3BP@CM NPs, prolongs blood circulation time while effectively blocking CD47 on CRC cells’ surface membranes. This leads to increased macrophage phagocytosis of CRC cells. Additionally, MPB-3BP@CM NPs promote apoptosis in CRC cancer by inhibiting glycolysis through blocking energy supply pathways (93).

## 5 The advantages and disadvantages of our research

This study is the first to use bibliometrics to provide a comprehensive description of CRC and nanomedicine, helping scholars in the field to grasp the hot spots, priorities, and frontiers of the research. However the study had some limitations. First of all, our research literature is entirely from WOSCC, which may lead to the omission of some literature, but this is a shortcoming of all bibliometrics methods, WOSCC is a comprehensive and authoritative database, and we are confident that it can reflect the state of the research field. Second, we cannot ensure that every document included in the study fully meets the search requirements. Finally, the quality difference of the literature may have some influence on the results.

## 6 Conclusion

This study used bibliometrics and visual analysis to quantify 12 years of research on CRC and nanomedicine in the WOSCC database. The top three countries contributing to CRC and nanomedicine are China, the United States, and India. At the same time, the study found that the number of CRC cases and deaths is rising in low - and middle-income countries while remaining stable in developed countries. In summary, the hot topics of CRC and nanomedicine research include:1. The significance of NPs in targeted therapy for CRC. 2. NDDS can promote targeted drug delivery and optimize therapeutic effects. Emerging trends in this area of research include: 1. PDT is a minimally invasive method for the death and destruction of CRC tumor cells, and NPs can promote the absorption of PS by tumor cells. 2. NPs with ICD-inducing properties is regarded as an effective method to combat the immune evasion of cancer cells and inhibit cancer metastasis, and have been actively applied in the treatment of CRC. 3. The use of NPs to solve the difficulties of TEM in the treatment of CRC has practical clinical significance. 4. Folate-modified NPs and Ph-dependent NPs are at the forefront of future developments in this field. This timely review explores trends and hot spots in CRC and nanomedicine research that could advance the field.

## Data Availability

The original contributions presented in the study are included in the article/supplementary material. Further inquiries can be directed to the corresponding author.
